# Design of a Supraharmonic Monitoring System Based on an FPGA Device

**DOI:** 10.3390/s22052027

**Published:** 2022-03-04

**Authors:** Dimitris A. Barkas, George Ch. Ioannidis, Stavros D. Kaminaris, Constantinos S. Psomopoulos

**Affiliations:** Department of Electrical & Electronics Engineering, University of West Attica, 250 Thivon Av., 122 44 Aegaleo, Greece; gioan@uniwa.gr (G.C.I.); skamin@uniwa.gr (S.D.K.)

**Keywords:** supraharmonics, FPGA, VHDL, monitoring, advanced power measurements, Fourier analysis

## Abstract

During the last few decades, the poor quality of produced electric power is a key factor that has affected the operation of critical electrical infrastructure such as high-voltage equipment. This type of equipment exhibits multiple different failures, which originate from the poor electric power quality. This phenomenon is basically due to the utilization of high-frequency switching devices that operate over modern electrical generation systems, such as PV inverters. The conduction of significant values of electric currents at high frequencies in the range of 2 to 150 kHz can be destructive for electrical and electronic equipment and should be measured. However, the measuring devices that have the ability of analyzing a signal in the frequency domain present the ability of analyzing up to 2.5 kHz–3 kHz, which are frequencies too low in comparison to the high switching frequencies that inverters, for example, work. Electric currents at 16 kHz were successfully measured on an 8 kWp roof PV generator. This paper presents a fast-developed modern measuring system, using a field programmable gate array, aiming to detect electric currents at high frequencies, with a capability for working up to 150 kHz. The system was tested in the laboratory, and the results are satisfactory.

## 1. Introduction

Electric power plays a key role in shaping the new trends in the way of life of modern man. This means that the corresponding power quality should be of a high level, and this is important in order to ensure reliable electrical grids with zero blackouts and failures of critical infrastructure. For this reason, it is critical to monitor the quality of the produced electric current, especially when the production embeds power electronics. Power electronics can operate under a wide range of switching conditions, starting at about 1 kHz and going up to several megahertz [1]. The requirement for higher-efficiency electrical grids and lower emissions of traditional power frequency harmonics has led to the emission of frequency components in the range of 2 kHz to 150 kHz [2,3,4].

Supraharmonics are frequency components located in the frequency range of 2 to 150 kHz, and they have not been examined as thoroughly as other power-quality issues due to a variety of factors. The lack of trustworthy measurement technology and widely established data analysis procedures has resulted in harmonics in that range not being able to be effectively documented in the past. More importantly, researchers were uninterested in attenuation because no severe interference issues had been linked to them. However, there has recently been a surge in research in that area. Improved measurement methods that can operate satisfactorily in this frequency range have been proposed [5,6]. The widespread use of this frequency range for powerline communication (PLC) has prompted worries about possible interferences that could jeopardize critical smart-grid services such as two-way communication and smart-meter readings. There have also been reports of negative impacts on electronic devices in the proximity of supraharmonic sources. Devices using shunt capacitors typically have lower impedance than the grid at this frequency range, providing a better channel for supraharmonic currents. The resultant currents can cause electrolytic capacitors to lose a large amount of charge and shorten their lifespan. Supraharmonics have also been linked to electrical equipment faults and unpredictable behavior, noise caused by mechanical resonance excitation, the tripping of residual current devices and LED lighting flickering [7,8,9,10,11].

**Figure 1:**
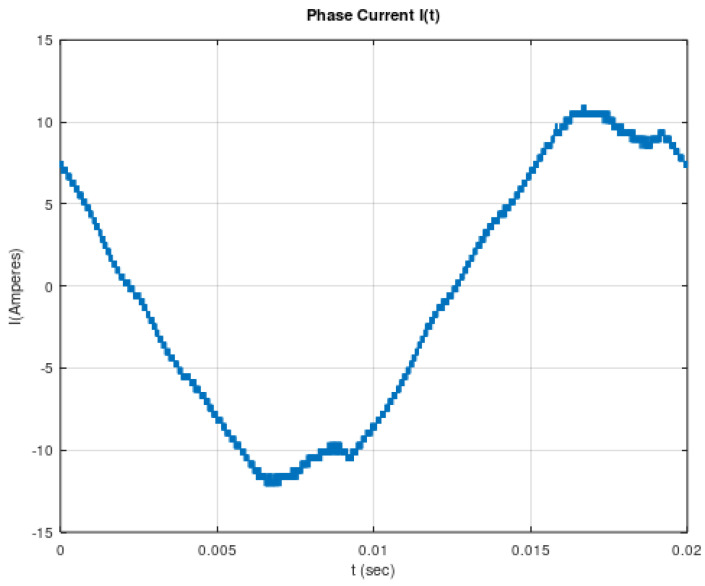
Measured electric current at the AC side of the inverter, from an 8 kWp roof PV generator.

**Figure 2:**
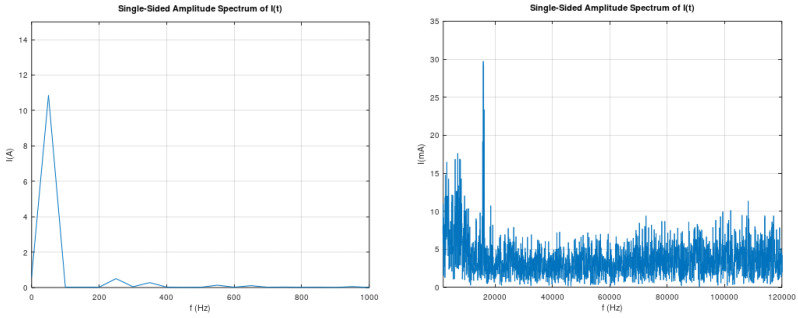
Fourier analysis of electric current at the AC side of the inverter from an 8 kWp roof PV generator.

A characteristic example of devices that produce electric currents with frequencies in the range of supraharmonics are inverters. Inverters are employed to convert DC to AC electric power, as in the case of photovoltaic (PV) systems. Electric-current quality has been able to be monitored and analyzed up to about 3 kHz until the present, due to the lack of knowledge on how supraharmonics could affect the power quality and the critical infrastructure [12,13]. The existence of supraharmonics in low-voltage networks, due to power electronics, was verified by the authors through measurements in an 8 kWp rooftop PV installation. The initial measuring system was simple enough, as it consisted of a digital oscilloscope (Gwinstek GDS-^210^2 A) in combination with a current probe (ELDITEST CP6220). These measurements verified the existence of supraharmonics on the current waveform at the AC side of the inverter. Figure 1 illustrates the measured electric current at the AC side of the inverter, while Figure 2 illustrates the analysis in the frequency domain for this measured current. From Figure 2, it is observed that the electric current at 50 Hz (the basic power-grid frequency) is about 11 A, while at 16 kHz, the corresponding current is about 30 mA, which imposes a harmonic distortion due to the 16 kHz component, of 0.27%.

The existence of supraharmonics has received high, significant attention in recent years due to the continuously increasing use of power electronics in distributed systems and the effects that they cause. In this context, significant efforts globally exist, as CENELEC has addressed the issue of the potential threat to networks and devices, as well as in accurately measuring energy. The European Association of National Metrology Institutes (EURAMET) is already conducting significant work on developing measuring methods, as the supraharmonics are identified as one of the key parameters that affect the reliable operation of smart grids [3,4]. Based on these, it is obvious that there is a need for developing and using advanced measuring devices for detecting and analyzing supraharmonics.

The purpose of this work was the development of an FPGA (field programmable gate array)-based supraharmonic measurement system, which uses a hardware description language for the control of an analog-to-digital converter, and a graphical programming language, called LabView, for the collection and the analysis of the measurement data. The FPGA-based device used for this measurement system is a programmable device that contains an FPGA and some other peripherals, while its programming can be implemented using the graphical language LabView, offering, however, the ability to develop extra coding using the C and VHDL (VHSIC Hardware Description Language, where VHSIC means Very High-Speed Integrated Circuits Program) languages. This device was selected for its advantages, some of which are:

➢ Fast hardware development;
➢ Fast, easy and ready-to-use frequency-analysis tools;
➢ Multiple peripherals with easy programmability, such as Wi-Fi connectivity for online monitoring applications;
➢ High flexibility for future optimizations and developments, even in remote mode.

The manuscript is structured in four main sections. The Materials and Methods paragraph describes the mathematical tools that were critical for the selection of the main parameters for the design of the proposed measuring system, the sampling methods and hardware that were used for the sampling process, and the main FPGA (field programmable gate area) device. In the third main section, the Discussion section, there is a short discussion about the results. In the last section, some conclusions about the proposed measuring system, its flexibility and future optimizations are described.

## 2. Materials and Methods

### 2.1. Mathematical Description

The developed system was tested and confirmed in Greece, where the fundamental frequency of the electric grid is 50 Hz. However, the system is capable of measuring and analyzing electric current at grids where the fundamental frequency is 60 Hz, such as in the USA and Canada. This capability can easily be achieved, as the system was parametrically designed.

It is well known that an analog periodic signal can be represented by Fourier series analysis, which is the summation of a theoretically infinite number of sinusoidal signals at different frequencies (multiples of the fundamental frequency of the signal) and with different appropriate amplitudes. These sinusoidal signals are the so-called harmonics and constitute the frequency spectrum of the analog signal. On the other hand, the measurement of an unknown analog signal can be used to determine its frequency spectrum using mathematical analysis tools such as the Fourier transform (FT).

A single sinusoidal signal can be mathematically described in the time domain, through the following Equation (1) [14]:

(1) $$I\left(t\right)=A\;\cdot \;Re\{e^{j\;\cdot \;2\;\cdot \;\pi \;\cdot \;f\;\cdot \;t}\}$$

where the “*Re*” stands for the real part of the exponential factor, “*f*” stands for the frequency of the sinusoidal signal (50 Hz for the Greek electric network), and “*A*” stands for the amplitude of the sinusoidal signal.

The same signal can also be presented in the frequency domain as described below [15]:

(2) I(f)=2 · A · δ(ω−2 · π · f)

where “*ω*” is the angular frequency and “*δ*” stands for the Dirac delta function.

The Fourier transform can be applied on analog and digital signals. For the case of analog signals, the continuous time FT is used, and it is calculated using the following general formula [15,16]:

(3) $$F\left(j\omega \right)=\int_{-\infty }^{+\infty }f\left(t\right)\;\cdot \;e^{-j\omega t}\;dt$$

**Figure 3:**
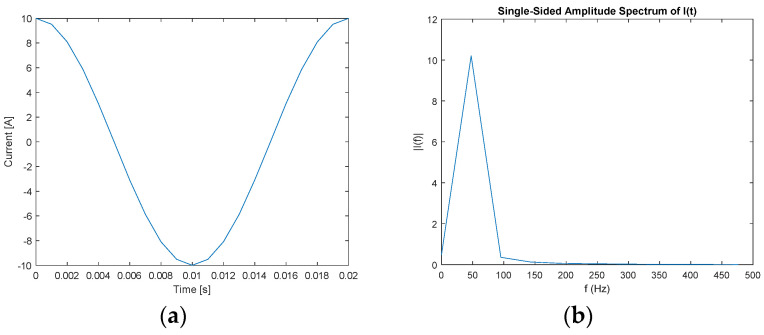
An example of sinusoidal signal in (**a**) time domain and (**b**) frequency domain.

An example of using FT on a sinusoidal signal for an electric current at 50 Hz with an amplitude of 10 A is illustrated in Figure 3.

The previously mentioned continuous FT can be applied on continuous (analog) signals in an infinite time slot, as Equation (3) describes. However, the implementation of measuring devices with the ability of analog signal analysis in real time is a difficult process and sometimes impossible. For this reason, the majority of measuring systems initially convert the analog signal into a digital signal. For the analysis of digital signals in the frequency domain, another tool called the discrete time Fourier transform (DTFT) is used, and the conversion from the time domain to the frequency domain can be achieved using the following Equation (4) [15]:

(4) $$X\left(\omega \right)=\overset{+\infty }{\underset{n=-\infty }{\sum }}\left\{x\left[n\right]\;\cdot \;e^{-jn\omega }\right\}$$

where *x*[*n*] are the samples over the analog signal *x*(*t*) and “*ω*” stands for the angular frequency.

Even if Equation (4) is capable of accurately calculating the frequency spectrum of the signal, a specific technical problem arises. This equation needs an infinite number of samples to calculate the frequency spectrum. To overcome the previously mentioned problem, another tool is used, called the discrete Fourier transform (DFT), which is expressed by the following equation [15,17]:

(5) $$X\left(k\right)=\overset{N-1}{\underset{n=0}{\sum }}\left\{x\left[n\right]\;\cdot \;e^{-jn\omega }\right\}$$

The difference between the DTFT and DFT is the limits on the sum factor. The DFT restricts the limits in a finite number of *N* samples. In Equation (5), the angular frequency is calculated using the formula [15]:

(6) $$\omega =\frac{2\;\cdot \;\pi \;\cdot \;k}{N}$$

where the domain *k* Є [0~*N* − 1].

The DFT mathematical tool is a strong tool in digital signal analysis with very reliable results. However, due to its complexity and the number of additions and multiplications needed to calculate the frequency spectrum, the DFT is a slow process in calculations, even on modern computers, and also difficult to implement on hardware. For this reason, some basic algorithms have been developed, resulting in a reduction in calculation complexity. These algorithms are known as fast Fourier transform (FFT) algorithms.

The FFT algorithms are based on a strategy called “divide and conquer”. This means that an *N*-point DFT can be split into two smaller DFTs of *N*/2 each. The calculation of the *N*-point DFT would require *N*^2^ additions and *N*^2^ multiplications. An *N*/2-point DFT requires (*N*/2)^2^ additions and (*N*/2)^2^ multiplications, which leads to reducing the calculation complexity. The two *N*/2-point DFTs can also be split into *N*/4-point DFTs each, etc. [15]. In this way, the complexity of the total calculations can be reduced, and finally, the frequency spectrum of a signal can be represented with less cost in hardware and software.

The two basic algorithm categories are [15]:

➢ Decimation-in-time FFT;
➢ Decimation-in-frequency FFT.

**Figure 4:**
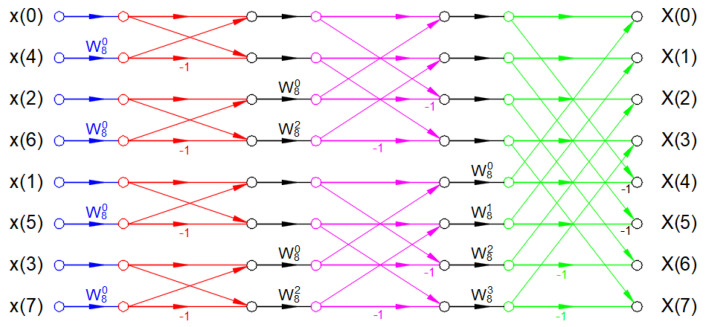
Eight-point radix-2 decimation-in-time FFT [15].

**Figure 5:**
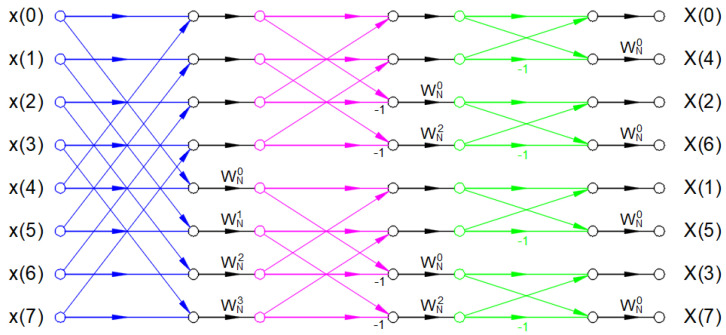
Eight-point radix-2 decimation-in-frequency FFT [15].

The usual implementation of FFT is the selection of an even number of *N* and also requiring the *N* to be a power of 2 (e.g., *n* = 1024 or 2^10^). Figure 4 graphically illustrates the implementation of an 8-point radix-2 decimation-in-time FFT algorithm, while Figure 5 illustrates the 8-point radix-2 decimation-in-frequency FFT algorithm.

The signals that exist in the electricity networks present significant periodic behavior, like many other electrical signals. These signals can be analyzed as several signals consisting of basic periodic functions, commonly sines and cosines. The standard Fourier transform is the mathematical method and the tool that allows the decomposition of the measured waveforms in these components. Every measured signal that is processed should have a finite size, to reduce the complexity and the required sources for the decomposition. The size of the sampled signal area, even though it is variable based on the signal, must be limited. The harmonic analysis is of paramount importance to detect, identify and evaluate different critical incidents, through the current and voltage waveforms. The detectability of specific signals in the presence of other nearby strong ones, the resolvability of nearby signals of similar strength and shifting ones, and biases in estimating the parameters of any of the aforementioned signals are all factors to consider [18,19].

In Figure 4 and Figure 5, the factors *W_N_^nk^* can be calculated using the following Equation (7) [16]:

(7) $$W_{N}^{nk}=e^{-\frac{2\;\cdot \;j\;\cdot \;\pi }{Ν}\;\cdot \;n\;\cdot \;k}$$

**Figure 6:**
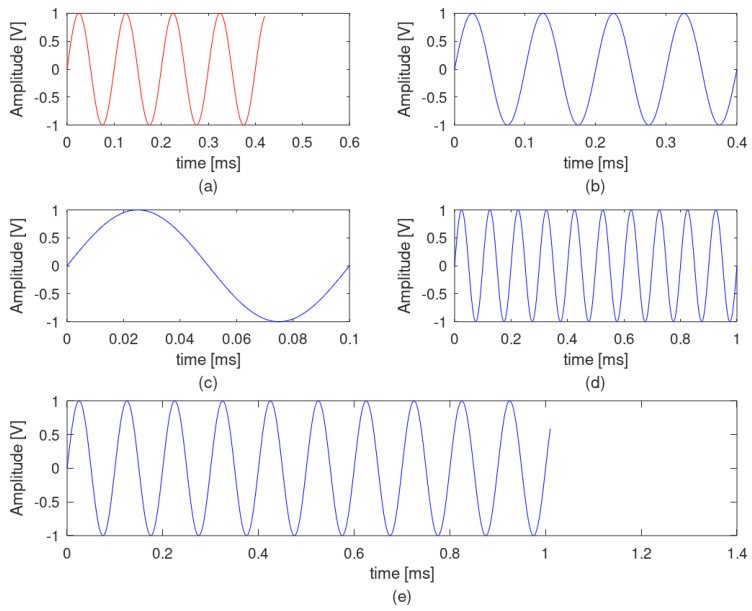
Example of different observation windows of the same sinusoidal signal at 10 kHz in the time domain. Observation time interval includes (**a**) four full periods of 10 kHz signal, but it is not an integer multiple of the 10 kHz signal period (0.42 ms); (**b**) exactly four full periods of 10 kHz signal (0.4 ms); (**c**) exactly one full period of 10 kHz signal (0.1 ms); (**d**) exactly ten full periods of 10 kHz signal (1 ms); and (**e**) ten full periods of 10 kHz signal, but it is not an integer multiple of the 10 kHz signal period (1.01 ms).

**Figure 7:**
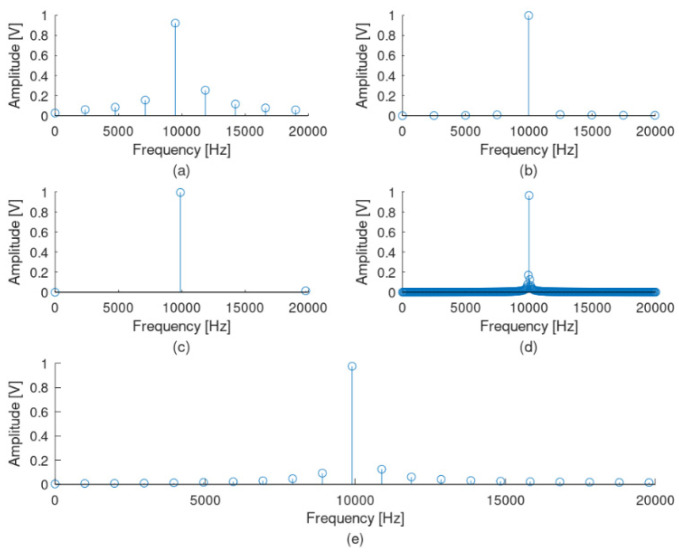
FFT results of the same sine signal at 10 kHz in the frequency domain, when different observation windows at the time domain are used. Observation time interval includes (**a**) four full periods of 10 kHz signal, but it is not an integer multiple of the 10 kHz signal period (0.42 ms); (**b**) exactly four full periods of 10 kHz signal (0.4 ms); (**c**) exactly one full period of 10 kHz signal (0.1 ms); (**d**) exactly ten full periods of 10 kHz signal (1 ms); and (**e**) ten full periods of 10 kHz signal, but it is not an integer multiple of the10 kHz signal period (1.01 ms).

Τhe selection of the signal’s observation interval is of paramount importance for the correct application of the FFT transformation. When the signal observation interval is not an integral propagation of a sinusoidal signal that will be measured, this leads to an FFT result spreading out in the frequency domain, leading to a well-known phenomenon called spectral leakage. As an example, a sinusoidal signal with an amplitude of 1 volt and a frequency of 10 kHz, sampled at a frequency of 1 MHz, is considered. Figure 6 presents this signal with five different observation time intervals, while Figure 7 presents the corresponding FFT results. In the first case (Figure 6a), the observation time interval is not an integer multiple of the 10 kHz period time, but this observation interval includes four full 10 kHz periods. The corresponding FFT (Figure 7a) gives a significant spread around the 10 kHz frequency, while this spread is not symmetric around the basic signal of 10 kHz. In a second case (Figure 6b), the observation time interval is an integer multiple of the 10 kHz signal period and includes exactly four full periods. The corresponding FFT (Figure 7b) is a single tone at 10 kHz. In a third case (Figure 6c), an exactly full period of 10 kHz is used as an observation interval, resulting in an also single tone as the FFT result (Figure 7c). In a fourth case, the observation interval is increased at exactly ten full periods, resulting in a better FFT result with less symmetric spread (Figure 6 and Figure 7d). Finally, a case with no integer multiple of the 10 kHz signal period including ten full periods is examined (Figure 6e). In this case, the FFT results in a non-symmetric spread of frequencies around the frequency of 10 kHz (Figure 7e). However, Figure 6 and Figure 7 refer to the recognition of a specific frequency, a situation that is not the usual case when real signals are measured. In reality, such systems aim to recognize a band of frequencies, such as the band of supraharmonics described in this manuscript. That means that the spectral leakage cannot be zeroed but only limited. This is where the so-called windows are introduced in such signal-processing applications. Windows are data-weighting functions used to limit the spectral leakage caused by finite observation intervals. Many window examples are well known [18].

**Figure 8:**
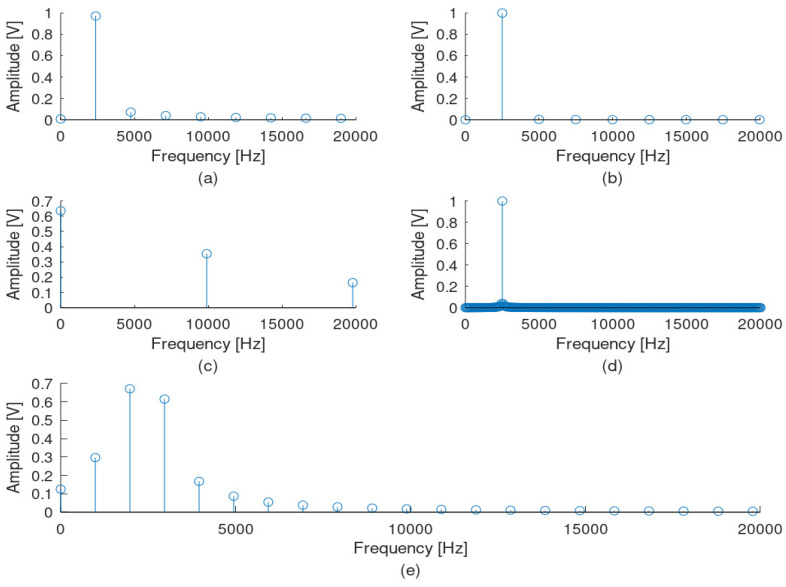
FFT results for the sine signal at 2.5 kHz in the frequency domain, when different observation windows at the time domain are used. Observation time intervals are (**a**) 0.42 ms, (**b**) 0.4 ms, (**c**) 0.1 ms, (**d**) 1 ms and (**e**) 1.01 ms.

**Figure 9:**
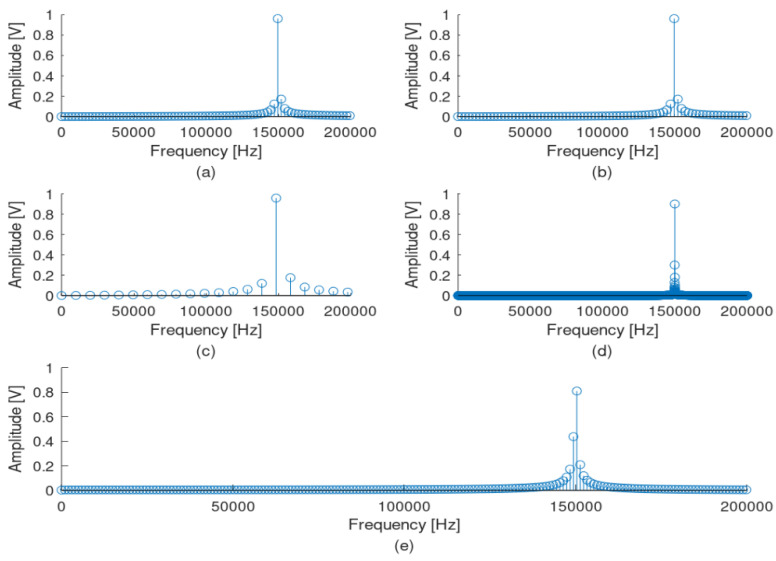
FFT results of the sine signal at 150 kHz in the frequency domain, when different observation windows at the time domain are used. Observation time intervals are (**a**) 0.42 ms, (**b**) 0.4 ms, (**c**) 0.1 ms, (**d**) 1 ms and (**e**) 1.01 ms.

**Figure 10:**
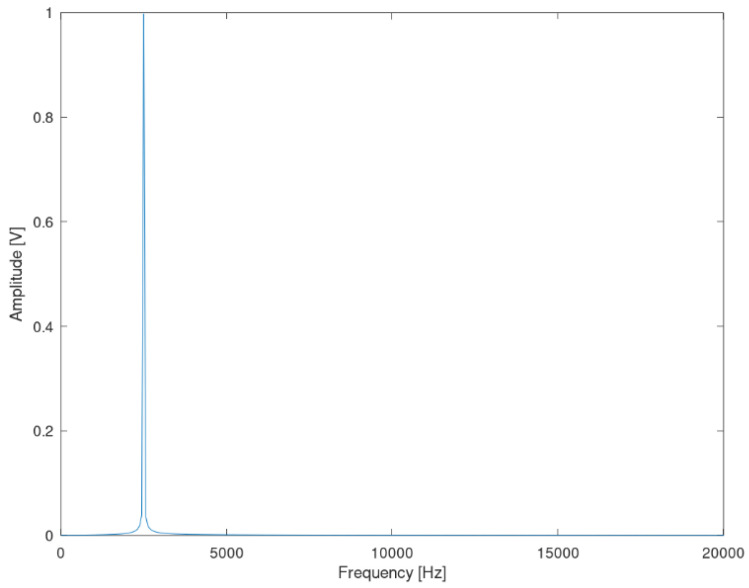
FFT results of the sinusoidal signal at 2.5 kHz in the frequency domain, using 16,384 samples at 1 MHz sampling rate (16.384 ms).

This research examined the measurement and the recognition of frequencies in the supraharmonic band without using a specific window technique but only enlarging the observation time, using a new measuring and signal analysis device based on FPGAs. This approach seems to limit the spectral leakage of the FFT result but with a negative impact on the hardware throughput. This issue is expected to be solved during the further development of the measuring system, as additional functionalities and improvements will be added, including some windowing techniques and following previous work on the topic, such as the ones proposed by Sandrolini and Mariscotti [18]. Figure 8, Figure 9 and Figure 10 prove the limited spectral leakage observed during the signal simulated procedures.

Figure 8 illustrates the FFT result of a 2.5 kHz sinusoidal signal observed at the same time intervals as the one used for the 10 kHz mentioned above. Figure 9 presents the corresponding FFT results for a 150 kHz sinusoidal signal also observed at the same time intervals. From the three mentioned cases, it is obvious that, the higher the observation time interval is (for the same sampling rate of 1 Msps), the lower the spectral leakage for the supraharmonic area becomes. In these cases, the best results correspond to a total observation time interval of 1 ms (ten full periods of the 10 kHz signal). In the proposed system, a total number of 16,384 samples are captured with a sampling rate of 1 MHz, leading to a very high observation time of 16,384 ms. Figure 10 illustrates the result of the FFT for the worst case of 2.5 kHz (the minimum frequency in the supraharmonic band) for a 16.384 ms observation time. From Figure 8, it seems that this number of samples is sufficiently high for the aims of the designed system.

### 2.2. The Sampling Method

**Figure 11:**
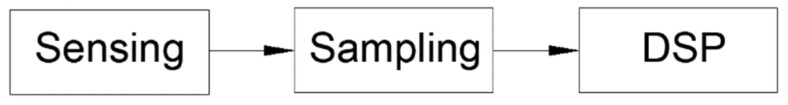
Basic steps of a digital measuring system using FFT.

Sampling is the first step in the analysis chain for an analog signal. Figure 11 presents the basic steps for analyzing an analog signal. Firstly, a sensing element is required. If the aim is the analysis of the electric current flowing in a conductor, then the sensing element could be a shunt resistor or a measuring current transformer. The analog signal is then sampled and converted into a digital one. Finally, the digital signal is mathematically processed using a digital signal processor (DSP) to implement the FFT calculation.

The aim of the system was the ability to sample and analyze analog signals with a frequency spectrum from 2.5 kHz up to 150 kHz. According to the Nyquist sampling theorem, the sampling frequency must be at least twice the maximum frequency that is contained in the signal and needs to be measured [15,20,21]:

(8) $$F_{S}\geq 2\;\cdot f_{max}$$

**Figure 12:**
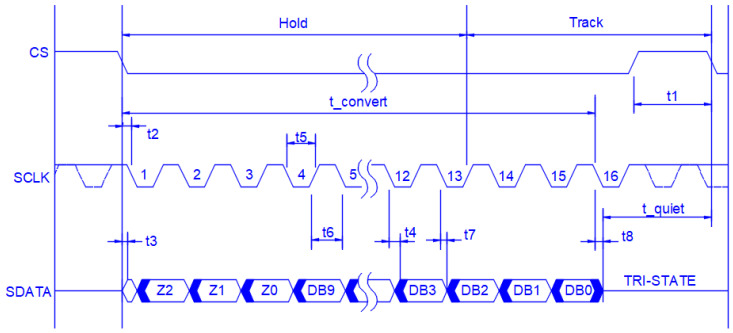
Serial interface timing diagram for the ADCS7476 [22].

The sampling frequency is a critical factor of the sampling system because it helps with the best representation of the initial signal after the sampling process. However, there should be a balance between better representation and the cost of implementation. For this reason, the sampling systems usually work with a sampling frequency 8–10 times the maximum frequency that needs to be captured. The aim of the developed system is the capture of analog signals with frequency components from 2.5 kHz up to 150 kHz. This requirement results in a sampling frequency of 1 MHz (1 Msps). The selected ADC, named ADCS7476, is a positive unipolar analog-to-digital converter, which means that it can work by pairing with sensors with positive voltage outputs (e.g., 0–5 V and 0–10 V). The maximum input signal should not exceed the operation voltage of the ADC, which is a maximum of 5 V. If the sensor outputs a signal over 5 V, then an appropriate voltage divider should be inserted into the total measuring system. The selected ADC can sample up to 1 Msps, with 1 Msps achieved when the clock signal on the ADC chip is set at 20 MHz. Moreover, the ADC can have a resolution of up to 10 bits. The control of this ADC was implemented using its serial interface, with the specifications that are presented in Figure 12, while Table 1 presents the timing requirements.

### 2.3. The FPGA-Based Device

The control of the ADC and the FFT implementation were developed on an FPGA-based device called MyRio. The embedded FPGA in this device is the Xilinx Zynq-7010 [23,24]. This device is capable of being programmed using the LabVIEW graphical language and supports the import of VHDL codes. VHDL code is a type of hardware description language. It is used for the description of hardware structure over an FPGA or a complex programmable logic device (CPLD) [25].

**Figure 13:**
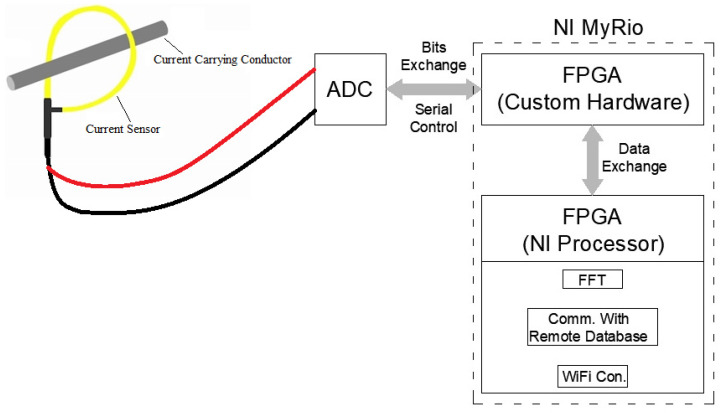
Proposed and implemented measuring system using the MyRio device and external ADC.

**Figure 14:**
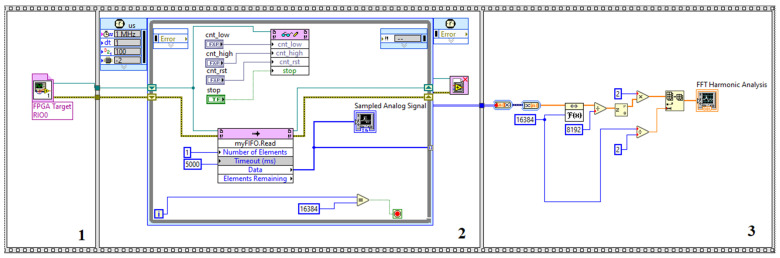
LabView code used for the design of the proposed measurement system. **1**. First frame of the code embeds the VHDL code; **2**. Second frame is a loop structure with 1MHz clock; **3**. Third frame is the FFT analysis.

**Figure 15:**
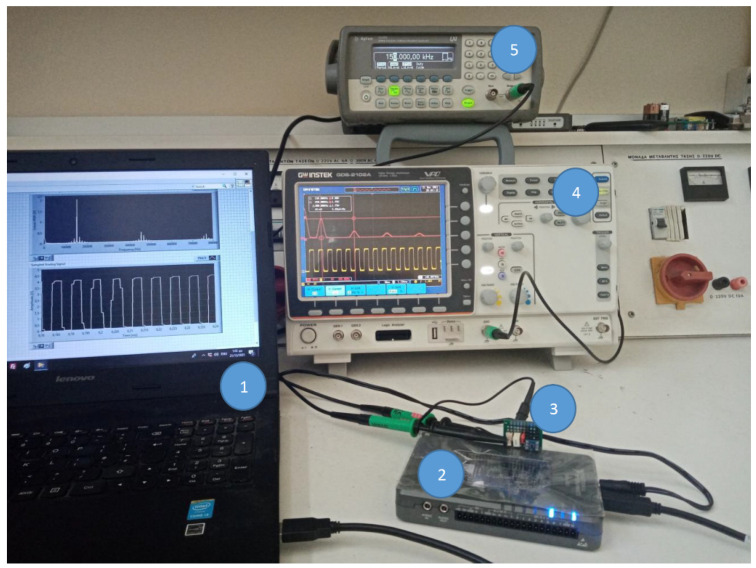
Experimental setup for the tested system. (**1**) PC screen; (**2**) MyRio device; (**3**) Custom ADC card; (**4**) Oscilloscope; (**5**) Pulse generator.

**Figure 16:**
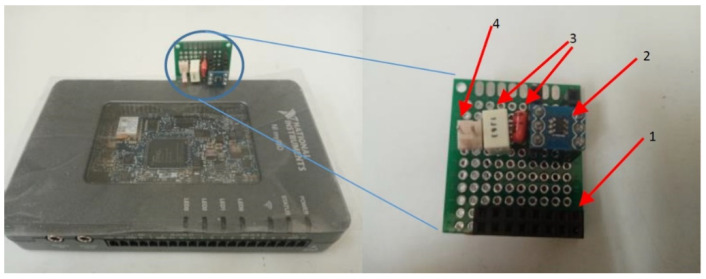
Custom ADC card attached on the MyRio device. (**1**) Bus connector; (**2**) ADC chip; (**3**) Coupling capacitors; (**4**) Input signal.

Figure 13 illustrates the general structure of the measuring device. The programming structure for the developed measuring system embeds two programming parts. The first part, which is a VDHL code, controls the external ADC, and the second part, which is a LabVIEW code, implements the FFT analysis and embeds the VDHL code. The LabVIEW code runs over a processor manufactured by National Instruments (NI), which is, by default, loaded on the FPGA, while the VDHL code runs directly over the FPGA. Figure 14 presents the LabView code that implements the proposed measurement system. The code is constituted by three frames. In the first frame, the LabView code embeds the VHDL code. In the second frame, a loop structure with a clock of 1 MHz runs continuously. During this frame, there is an exchange of commands and data to and from the ADC through a bus connection. A FIFO (first in–first out) memory manipulates the data from the ADC. The last frame implements the FFT analysis. Figure 15 illustrates the lab setup. In this setup, a USB cable connects the PC with the MyRio device to display the FFT analysis result on its screen. The MyRio device controls the ADC chip through an SPI-based serial interface, while a pulse generator feeds the analog input of the ADC. Figure 16 presents the manufactured custom ADC card.

The NI processor, located in the FPGA, implemented the FFT analysis. In the frame of the presented paper, the FFT calculation used the embedded FFT function that LabView offers. However, an optimized approach for the FFT implementation can be achieved, using already-proposed approaches [25,26].

**Figure 17:**
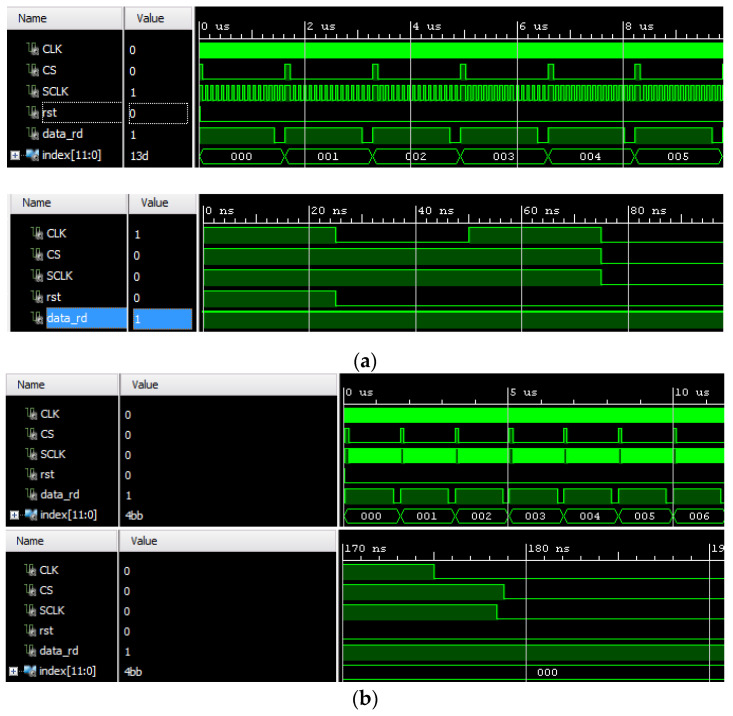
Simulation of VHDL code. (**a**) Prior synthesis process; (**b**) After synthesis process.

The ADC control was described in VDHL as already mentioned. The ADC resolution, which can be up to 10 bits, was VDHL parametric, and the developed system set up used a 10-bit resolution for test purposes. That means that 5 V of measured signal represented a value of 2^10^ − 1 = 1023. Figure 17 illustrates the simulation results for the VDHL code that controlled the external ADC.

## 3. Results

The tests for the developed system were performed in the High Voltage Laboratory of the University of West Attica in Greece. The ADC resolution was 10 bits, which means a maximum decimal value of 1023.

The confirmation of the measurements was conducted using specific and predefined signals produced by a signal generator, and with the use of a calibrated oscilloscope (Gwinstek GDS-2102 A). These selected signals were:

➢ Sinusoidal signal 5 V peak–peak at 15 kHz (offset = 2.5 VDC);
➢ Sinusoidal signal 5 V peak–peak at 60 kHz (offset = 2.5 VDC);
➢ Sinusoidal signal 5 V peak–peak at 150 kHz (offset = 2.5 VDC);
➢ Sinusoidal signal 2 V peak–peak at 15 kHz (offset = 1 VDC);
➢ Sinusoidal signal 2 V peak–peak at 60 kHz (offset = 1 VDC);
➢ Sinusoidal signal 2 V peak–peak at 150 kHz (offset = 1 VDC);
➢ Rectangular signal 5 V peak–peak at 15 kHz fundamental frequency (offset = 2.5 VDC);
➢ Rectangular signal 5 V peak–peak at 60 kHz fundamental frequency (offset = 2.5 VDC);
➢ Rectangular signal 5 V peak–peak at 150 kHz fundamental frequency (offset = 2.5 VDC);
➢ Rectangular signal 2 V peak–peak at 15 kHz fundamental frequency (offset = 1 VDC);
➢ Rectangular signal 2 V peak–peak at 60 kHz fundamental frequency (offset = 1 VDC);
➢ Rectangular signal 2 V peak–peak at 150 kHz fundamental frequency (offset = 1 VDC).

**Figure 18:**
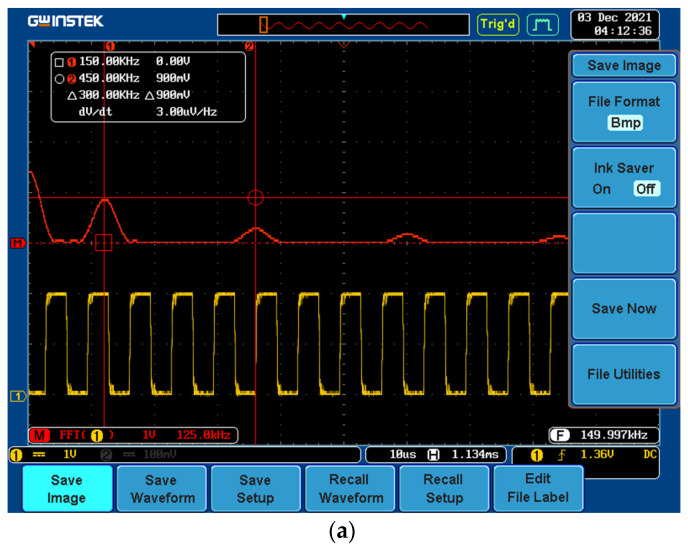

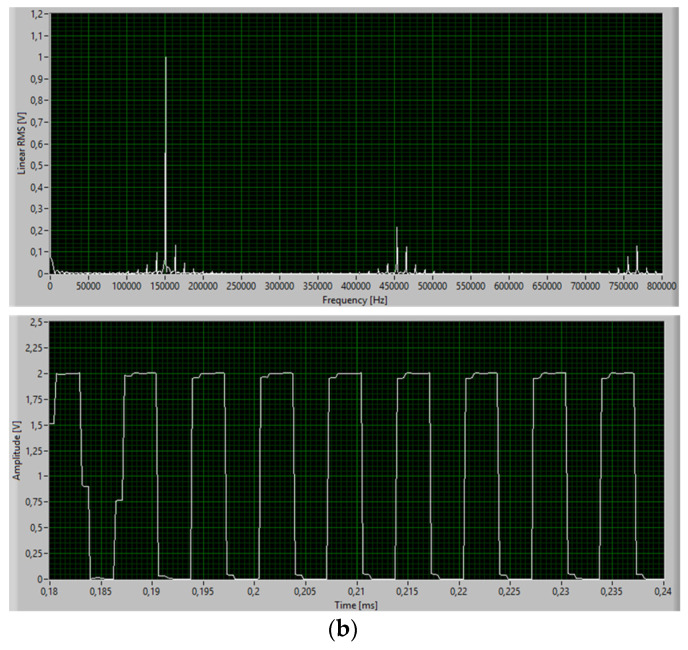
Tested rectangular signal with amplitude 2 Vp-p and offset 1 VDC, with fundamental frequency of 150 kHz. (**a**) Measured using a calibrated oscilloscope (Gwinstek GDS-2102A); (**b**) Measured using the custom FPGA-based system.

Figure 18 presents the measured worst-case scenario, where a 150 kHz fundamental frequency 2 Vp-p with a 1 VDC offset signal was attached on the ADC card. Corresponding measurements were performed for the cases mentioned above. Table 2, Table 3, Table 4, Table 5, Table 6, Table 7, Table 8 and Table 9 present the comparisons of the FPGA system to the calibrated oscilloscope.

Table 2, Table 3, Table 4, Table 5, Table 6, Table 7, Table 8 and Table 9 present the results of a comparison between the FPGA custom system and the oscilloscope. These tables include the proposed system frequency and amplitude measurements in columns one and three, respectively; the oscilloscope’s frequency and amplitude measurements in columns two and four, respectively; and the frequency error and amplitude error between the oscilloscope’s measurement and the proposed system’s measurement in columns five and six, respectively. The amplitude errors between the two kinds of measurement (FPGA system and oscilloscope) were calculated through Equation (9).

(9) $$\Delta V\left[\%\right]=\frac{SysV-SimV}{SimV}\;\cdot \;100\;$$

where *SysV* refers to the proposed measurement system measurement and *SimV* refers to the oscilloscope measurement.

Table 2 and Table 3 illustrate the comparisons between the FPGA system and the oscilloscope when the applied signal was sinusoidal with 5 Vpp (offset, 2.5 VDC) and 2 Vpp (offset, 1 VDC), respectively. From the comparisons, it is obvious that the FPGA system is capable of detecting, with high accuracy, the frequency values (as can be observed from the frequency difference, Δf(%), between the two measurement methods, the FPGA system and oscilloscope), while there is an error for the amplitude when the frequency approaches the high-frequency limit of the designed system (150 kHz).

Table 4, Table 5 and Table 6 present the results arising from the comparison between the FPGA system and the oscilloscope, after the application of rectangular signals with an amplitude of 5 Vpp (offset, 2.5 VDC) and with fundamental frequencies at 15 kHz, 60 kHz and 150 kHz, respectively. Correspondingly, Table 7, Table 8 and Table 9 present the measurements in rectangular signals with an amplitude of 2 Vpp (offset, 1 VDC) and with fundamental frequencies at 15 kHz, 60 kHz and 150 kHz, respectively. The FPGA system can detect, with high accuracy, the frequency values of the signal, while some errors for the amplitude values of each frequency component resulted. Additionally, when the fundamental frequency of the applied rectangular signal approaches the limit of the designed system (150 kHz), the errors become higher. There is also a significant error for the frequencies of 45 kHz, 75 kHz and 60 kHz when a rectangular signal is applied.

As can be seen from Table 4, Table 5, Table 6, Table 7, Table 8 and Table 9, there is a significant difference between the two measurement systems, the proposed system and the oscilloscope. This difference appears when the signal is not purely sinusoidal. This difference can be attributed to the following reasons:

1. The system is designed to measure harmonic signals with frequencies up to 150 kHz, namely, the supraharmonic higher frequencies, according to standards. Thus, any higher frequency is not measured and, consequently, signals presenting higher frequencies are not accurately measured.
2. Rectangular signals are the result of the sum of numerous harmonics, and the lower one is known as the fundamental frequency. Thus, by default, they include in their signals frequencies higher than the fundamental, resulting in the need for significantly higher sampling rates for accurate measurement.

As the proposed system was designed to measure up to 150 kHz, it was expected to measure all signals presenting harmonic content higher than 150 kHz with reduced accuracy. Especially for the proposed system, Table 10 presents the difference in the harmonic components of the measurements by the proposed system and oscilloscope for the rectangular signals used for the evaluation of the proposed system (with fundamental frequencies of 15 kHz, 60 kHz and 150 kHz). The results of this table verify that differences exist between the oscilloscope and FPGA-based measurement systems, and can explain the significant difference that appeared. This is the case for every system designed for measuring a specific bandwidth of frequencies, and it is common in power-quality measurement applications. The oscilloscope can recognize more orders of harmonics than the custom FPGA system. This is because the oscilloscope uses a sample rate of 2 GS/s, compared to the proposed system, which uses only 1 MS/s. Additionally, the oscilloscope uses a vertical resolution of 8 bits, while the FPGA system uses a corresponding resolution of 10 bits, making the FPGA system less prone to noise. However, the measurements were conducted in the laboratory, where the noise is assumed to be low enough, and the impact on the final error is assumed to be small enough.

In the presence of significant errors in the measurement band, an error calibration method was used. This method uses the interpolation polynomial process. For this reason, the mean value of the error for the measured frequencies up to 150 kHz was calculated. Through this mean value, the error calibration function was calculated as a function of the frequency:

$$e\left(f\right)=f^{5}\;\cdot \;1.3190\;\cdot \;10^{-22}-f^{4}\;\cdot \;4.5023\;\cdot \;10^{-17}+f^{3}\;\cdot \;5.1895\;\cdot \;10^{-12}-f^{2}\;\cdot \;2.3415\;\cdot \;10^{-7}+f\;\cdot \;3.5674\;\cdot \;10^{-3}-1.6521\;\cdot \;10^{1}$$

where *f* is the frequency in hertz at which the error was calculated.

Table 11 presents the errors before and after the calibration process for the case of the rectangular signals described above. The error correction refers to the frequency band that the system is capable of measuring. The only instance is the case of 105 kHz, where the error was increased. However, in all the other frequency areas, the error seems to have been decreased.

## 4. Discussion

The described system has been under development until now. This paper presents the more critical results in comparison to a real calibrated oscilloscope model, Gwinstek GDS-2102 A. The calculated errors in Table 2, Table 3, Table 4, Table 5, Table 6, Table 7, Table 8, Table 9, Table 10 and Table 11 confirm that the system can detect, with high accuracy, the frequency values of the applied signals. The system shows sufficient accuracy for the amplitude of each frequency component, even if, in a specific frequency range around 100 kHz, the error presented in the amplitude measurements seems to be increased. For the frequencies higher than 150 kHz, the error presented is expected, as the system is designed to accurately detect signals up to 150 kHz, which is the limit of the supraharmonic spectrum as defined by regulations. The results over this frequency level show that the system can detect frequencies with acceptable error. The error inside the supraharmonic spectrum could be suppressed using some kind of corrective factor or technique such as the polynomial regression as already described in the Results section. Moreover, this implementation used a general-purpose circuit board for the construction of the custom ADC card, which means that there was a high probability of external noise, as the frequencies are high enough and can create the phenomenon of an antenna for the wires [27], even if the custom manufactured ADC card was constructed as carefully as it could be, using techniques to decrease this kind of noise. Additionally, the use of a specific printed circuit board (PCB) is expected to further decrease this kind of noise.

Regarding hardware optimizations, one future improvement could be the introduction of an offset circuit in the signal input of the ADC card, or a dual-polarity ADC, to extend the sensor selection, for example, to select a sensor −5 to 5 V [28]. However, the use of a dual-polarity ADC is expected to create new hardware requirements such as additional power supplies.

## 5. Conclusions

This paper presents a measuring system aiming to analyze the frequency spectrum of analog signals in the range of 2.5 kHz–150 kHz. This system is capable of operating with sensors with outputs of 0–5 V. The insertion of additional hardware components can increase the range of applicable sensors. In this way, sensors with outputs other than 0–5 V, such as −10 to 10 V, will provide the system with modularity and easy expandability, which is an advantage in high-frequency measuring devices. The presented results confirm the capability of the system to estimate and present, with fair accuracy, the frequency spectrum of the measured signals. The system can operate successfully, while the flexibility of the system offers the chance to support more sensors that can measure signals in ranges between −10 and +10 V. Additionally, the design of an appropriate PCB can also improve the system’s efficiency when it operates in real industrial environments, because of a reduction in the produced electromagnetic interference.

## Figures and Tables

**Table 1:** Timing requirements for the ADCS7476 [22].

*Parameter*	*Description*	*Conditions*	*Min Value*	*Typical Value*	*Max Value*	*Units*
***t_CONVERT_***		Τ_A_ = 25 °C	16 × t_SCLK_			
***T_QUIET_***	*Quiet time*		50			ns
***t*_1_**	Minimum $\overset{ˉ}{\mathrm{CS}}$ pulse width		10			ns
***t*_2_**	$\overset{ˉ}{\mathrm{CS}}\;$to SCLK setup time		10			ns
***t*_3_**	Delay from $\overset{ˉ}{\mathrm{CS}}\;$until SDATA TRI-STATE disabled				20	ns
***t*_4_**	Data access time after SCLK falling edge	V_DD_ = 4.75~5.25 V			20	ns
***t*_5_**	SCLK low pulse width		0.4 × t_SCLK_			ns
***t*_6_**	SCLK high pulse width		0.4 × t_SCLK_			ns
***t*_7_**	SCLK to data valid hold time	V_DD_ = 4.75~5.25 V	5			ns
***t*_8_**	SCLK falling edge to SDATA high impedance	V_DD_ = 4.75~5.25 V	5		25	ns
***t_POWER-UP_***	Power-up time from full power down	Τ_A_ = 25 °C		1		μs

**Table 2:** Comparisons between the calibrated oscilloscope and the FPGA-based system measurements for sinusoidal signals with amplitude of 5 Vpp with 2.5 VDC offset value, for signal frequencies of 15 kHz, 60 kHz and 150 kHz.

Sinusoidal Signals with Amplitude 5 Vpp, Offset 2.5 VDC					
Frequency (System) (kHz)	Frequency (Oscilloscope) (kHz)	RMS Value (System) (V)	RMS Value (Oscilloscope) (V)	Δf (%)	ΔV (%)
15.00	15.00	1.80	1.80	0.00	0.00
60.00	60.00	1.87	1.80	0.00	−4.17
151.00	151.25	1.95	1.78	0.16	−9.55

**Table 3:** Comparisons between the calibrated oscilloscope and the FPGA-based system measurements for sinusoidal signals with amplitude of 2 Vpp with 1 VDC offset value, for signal frequencies of 15 kHz, 60 kHz and 150 kHz.

Sinusoidal Signals with Amplitude 2 Vpp, Offset 1 VDC					
Frequency (System) (kHz)	Frequency (Oscilloscope) (kHz)	RMS Value (System) (V)	RMS Value (Oscilloscope) (V)	Δf (%)	ΔV (%)
15.00	15.00	0.72	0.72	0.00	−0.28
60.50	61.25	0.76	0.77	1.22	0.78
150.75	150.00	0.79	0.70	−0.50	−12.86

**Table 4:** Comparisons between the calibrated oscilloscope and the FPGA-based system measurements for rectangular signal with amplitude of 5 Vpp with 2.5 VDC offset value, for a signal’s fundamental frequency at 15 kHz.

Rectangular Signal with Amplitude 5 Vpp, Offset 2.5 VDC @15 kHz Fundamental Frequency					
Harmonic Frequency (System) (kHz)	Harmonic Frequency (Oscilloscope) (kHz)	RMS Value (System) (V)	RMS Value (Oscilloscope) (V)	Δf (%)	ΔV (%)
15.00	15.00	2.25	2.21	0.00	−1.81
45.00	45.00	0.87	0.77	0.00	−14.23
75.00	75.00	0.39	0.45	0.00	12.22
105.50	106.00	0.35	0.33	0.47	−5.10

**Table 5:** Comparisons between the calibrated oscilloscope and the FPGA-based system measurements for rectangular signal with amplitude of 5 Vpp with 2.5 VDC offset value, for a signal’s fundamental frequency at 60 kHz.

Rectangular Signal with Amplitude 5 Vpp, Offset 2.5 VDC @60 kHz Fundamental Frequency					
Harmonic Frequency (System) (kHz)	Harmonic Frequency (Oscilloscope) (kHz)	RMS Value (System) (V)	RMS Value (Oscilloscope) (V)	Δf (%)	ΔV (%)
60.00	60.00	2.40	2.18	0.00	−10.09
180.00	180.62	0.77	0.77	0.34	−1.17
302.00	300.62	0.44	0.47	−0.46	5.58
422.50	420.62	0.27	0.30	−0.45	8.33

**Table 6:** Comparisons between the calibrated oscilloscope and the FPGA-based system measurements for rectangular signal with amplitude of 5 Vpp with 2.5 VDC offset value, for a signal’s fundamental frequency at 150 kHz.

Rectangular Signal with Amplitude 5 Vpp, Offset 2.5 VDC @150 kHz fundamental Frequency					
Harmonic Frequency (System) (kHz)	Harmonic Frequency (Oscilloscope) (kHz)	RMS Value (System) (V)	RMS Value (Oscilloscope) (V)	Δf (%)	ΔV (%)
150.00	150.00	2.50	2.18	0.00	−14.68
452.00	455.00	0.54	0.70	0.66	22.14

**Table 7:** Comparisons between the calibrated oscilloscope and the FPGA-based system measurements for rectangular signal with amplitude of 2 Vpp with 1 VDC offset value, for a signal’s fundamental frequency at 15 kHz.

Rectangular Signal with Amplitude 2 Vpp, Offset 1 VDC @15 kHz Fundamental Frequency					
Harmonic Frequency (System) (kHz)	Harmonic Frequency (Oscilloscope) (kHz)	RMS Value (System) (V)	RMS Value (Oscilloscope) (V)	Δf (%)	ΔV (%)
15.00	15.12	0.92	0.92	0.83	−0.44
45.00	45.00	0.36	0.30	0.00	−20.83
75.00	75.00	0.16	0.18	0.00	11.20
105.50	105.00	0.15	0.15	−0.48	0.00

**Table 8:** Comparisons between the calibrated oscilloscope and the FPGA-based system measurements for rectangular signal with amplitude of 2 Vpp with 1 VDC offset value, for a signal’s fundamental frequency at 60 kHz.

Rectangular Signal with Amplitude 2 Vpp, Offset 1 VDC @60 kHz Fundamental Frequency					
Harmonic Frequency (System) (kHz)	Harmonic Frequency (Oscilloscope) (kHz)	RMS Value (System) (V)	RMS Value (Oscilloscope) (V)	Δf (%)	ΔV (%)
60.00	60.00	0.96	0.90	0.00	−6.67
180.00	180.00	0.31	0.28	0.00	−8.13
302.00	300.00	0.17	0.18	−0.67	7.10
422.50	420.00	0.11	0.15	−0.59	26.67

**Table 9:** Comparisons between the calibrated oscilloscope and the FPGA-based system measurements for rectangular signal with amplitude of 2 Vpp with 1 VDC offset value, for a signal’s fundamental frequency at 150 kHz.

Rectangular Signal with Amplitude 2 Vpp, Offset 1 VDC @150 kHz Fundamental Frequency					
Harmonic Frequency (System) (kHz)	Harmonic Frequency (Oscilloscope) (kHz)	RMS Value (System) (V)	RMS Value (Oscilloscope) (V)	Δf (%)	ΔV (%)
150.00	150.00	1.00	0.90	0.00	−11.11
460.00	450.00	0.22	0.30	−2.22	26.67

**Table 10:** The frequency orders that the oscilloscope and the proposed system can recognize according to the fundamental frequency of an applied rectangular signal.

Fundamental Frequency (kHz)	Oscilloscope Order of Harmonics Measured	Proposed System Order of Harmonics Measured
15.00	*n* = 1, 3, 5, 7, 9, 11, 13	*n* = 1, 3, 5, 7
60.00	*n* = 1, 3, 5, 7, 9, 11	*n* = 1, 3, 5, 7
150.00	*n* = 1, 3, 5, 7, 9, 11, 13	*n* = 1, 3, 5

**Table 11:** The errors that occurred before and after the calibration process. ΔV1 refers to the error before the calibration. ΔV2 refers to the error after the calibration.

Rectangular Signal with Amplitude 5 Vpp, Offset 2.5 VDC @15 kHz Fundamental Frequency			Rectangular Signal with Amplitude 2 Vpp, Offset 1 VDC @15 kHz Fundamental Frequency		
Harmonic Frequency (System) (kHz)	ΔV1 (%)	ΔV2 (%)	Harmonic Frequency (System) (kHz)	ΔV1 (%)	ΔV2 (%)
15.00	−1.81	−1.44	15.00	−0.44	−0.08
45.00	−14.23	5.80	45.00	−20.83	0.35
75.00	12.22	1.94	75.00	11.20	0.80
105.50	−5.10	11.87	105.50	0.00	16.15
**Rectangular Signal with Amplitude 5 Vpp, Offset 2.5 VDC @60 kHz Fundamental Frequency**			**Rectangular Signal with Amplitude 2 Vpp, Offset 1VDC @60 kHz Fundamental Frequency**		
**Harmonic Frequency (System) (kHz)**	**ΔV1 (%)**	**ΔV2 (%)**	**Harmonic Frequency (System) (kHz)**	**ΔV1 (%)**	**ΔV2 (%)**
60.00	−10.09	−4.13	60.00	−6.67	−0.89
**Rectangular Signal with Amplitude 5 Vpp, Offset 2.5 VDC @150 kHz Fundamental Frequency**			**Rectangular Signal with Amplitude 2 Vpp, Offset 1 VDC @150 kHz Fundamental Frequency**		
**Harmonic Frequency (System) (kHz)**	**ΔV1 (%)**	**ΔV2 (%)**	**Harmonic Frequency (System) (kHz)**	**ΔV1 (%)**	**ΔV2 (%)**
150.00	−14.68	−0.94	150.00	−11.11	2.20
